# Surgery for chronic pancreatitis: the comparison of two high-volume centers reveals lack of a uniform operative management

**DOI:** 10.1007/s00423-021-02335-1

**Published:** 2021-10-01

**Authors:** Niccolò Surci, Claudio Bassi, Roberto Salvia, Giovanni Marchegiani, Luca Casetti, Giacomo Deiro, Christina Bergmann, Dietmar Tamandl, Martin Schindl, Jakob Mühlbacher, Klaus Sahora

**Affiliations:** 1 Department of Surgery, Vienna General Hospital, Medical University of Vienna, Vienna, Austria; 2 Department of General and Pancreatic Surgery, Pancreas Institute, “GB Rossi” Hospital, University and Hospital Trust of Verona, Verona, Italy; 3 Department of Radiology, Vienna General Hospital, Medical University of Vienna, Vienna, Austria

**Keywords:** Pancreatitis, Surgery, Timing, Clinicalmanagement, Guidelines

## Abstract

**Purpose:**

Many aspects of surgical therapy for chronic pancreatitis (CP), including the correct indication and timing, as well as the most appropriate operative techniques, are still a matter of debate in the surgical community and vary widely across different centers. The aim of the present study was to uncover and analyze these differences by comparing the experiences of two specialized surgical units in Italy and Austria.

**Methods:**

All patients operated for CP between 2000 and 2018 at the two centers involved were included in this retrospective analysis. Data regarding the clinical history and the pre- and perioperative surgical course were analyzed and compared between the two institutions.

**Results:**

Our analysis showed a progressive decrease in the annual rate of pancreatic surgical procedures performed for CP in Verona (no. = 91) over the last two decades (from 3% to less than 1%); by contrast, this percentage increased from 3 to 9% in Vienna (no. = 77) during the same time frame. Considerable differences were also detected with regard to the timing of surgery from the first diagnosis of CP — 4 years (IQR 5.5) in the Austrian series vs two (IQR 4.0) in the Italian series -, and of indications for surgery, with a 12% higher prevalence of groove pancreatitis among patients in the Verona cohort.

**Conclusion:**

The comparison of the surgical attitude towards CP between two surgical centers proved that a consistent approach to this pathology still is lacking. The identification of common guidelines and labels of surgical eligibility is advisable in order to avoid interinstitutional treatment disparities.

## Introduction

Chronic pancreatitis (CP) is an inflammatory condition leading to permanent structural damage of the pancreatic gland, with variable grades of impairment of its endocrine and exocrine functions [1]. The management of this pathology is challenging due to its multiple causes, natural history, impact on patients’ life, and potential complications [2]. Traditionally, the most commonly adopted management strategy is a multimodal step-up approach that includes behavioral-medical therapy, endoscopy, and - as salvage option for symptomatic refractory patients - operative intervention [3].

The first documented surgical experiences with chronic pancreatitis date back to the early twentieth century, when Gould (1898) and Moynihan (1902) detailed two successful procedures entailing the removal of calculi from the common pancreatic duct [4]. Since then, a growing number of increasingly complex and diverse surgical operations for the treatment of this disease were reported, until the description of the Puestow (1958) and the Partington-Rochelle (1960) drainage procedures, which represented two milestones in the history of surgery for CP [5, 6] . Operative procedures for chronic pancreatitis are currently grouped into decompression procedures (e.g., Puestow, Partington-Rochelle, Frey, and Beger techniques) and pancreatic resections (e.g., pancreatoduodenectomy, distal pancreatectomy, total pancreatectomy), whereas the previously described surgical denervation strategies have been largely abandoned because they have been proved to be ineffective [4, 5, 7]. The choice of surgical approach for CP should take into account the clinical characteristics and leading symptoms of patients, the anatomical status of the inflamed pancreatic gland (e.g., presence of stenosis, stones, dilation of the main pancreatic duct, parenchymal atrophy, inflammatory mass in the head of the gland), and the expertise of surgeons [8]. Furthermore, the considerable rate of postoperative morbidity and mortality (up to 60% and 4%, respectively) following pancreatic surgery - even if performed in highly specialized centers - calls for a careful, accurate, and preferably multidisciplinary selection of optimal candidates [9].

The aim of the present study is to describe, critically review, and compare the twenty years of experience with the surgical treatment of CP at two specialized surgical units in Italy and Austria.

## Materials and methods

This is a retrospective descriptive study involving two high-volume pancreatic centers - namely, the Department of Surgery of the Vienna General Hospital and the Department of General and Pancreatic Surgery of Verona—which established a scientific collaboration as part of a fellowship program for surgical residents. Patients treated for CP between 2000 and 2018 with either resecting (pancreatoduodenectomy, left pancreatectomy, total pancreatectomy) or decompressive surgical procedures (exclusively Frey or Partington-Rochelle lateral pancreatojejunostomy, according to the internal guidelines of the two institutions involved) were included in the study. The following additional inclusion criteria were defined: age ≥ 18 years, either sex; patients affected by CP (segmental or diffuse, groove pancreatitis) and treated via pancreatic resection or decompression procedure. Exclusion criteria included a histologically proven diagnosis of autoimmune pancreatitis, underlying occult pancreatic cancer at the time of surgery or presence of other malignant diseases. All data were obtained from the prospectively maintained patient registries of the two centers. This study was approved by the Ethics Committee of the two institutions concerned, namely the Ethics Committee of the University of Vienna (EK no. 1153/2020) and the Ethics Committee for Clinical Research of the provinces of Verona and Rovigo (protocol no. 1101CESC) and was performed in compliance with the Good Clinical Practice standard and the principles of the Declaration of Helsinki.

### Endpoints

The main aim of this retrospective analysis was to analyze and compare the surgical experience in the field of CP over the last twenty years focusing on the following topics: general patient characteristics, surgical approach, indications, and timing of intervention.

### Statistical analysis

No sample size calculation was performed due to the retrospective design of the study. Continuous variables are expressed as mean ± SD or as medians with interquartile range (IQR) as appropriate, whereas categorical variables are expressed as frequencies with percentages. For categorical data, the *χ*^2^ tests with Yates correction in 2–3–2 contingency tables was used. The t-Student paired test was used to compare mean values and, when appropriated, the Cohen’s D effect size was additionally calculated to describe the standardized differences between two means. The Wilcoxon test was used to compare medians. In general, a two-sided *p* value < 0.05 was considered as statistically significant. The statistical analysis was conducted using SPSS Statistic software version 26.0 (IBM Corporation, Armonk, NY, USA) and Stata 14.0 (Stata Corp, College Station, TX).

## Results

**Fig. 1:**
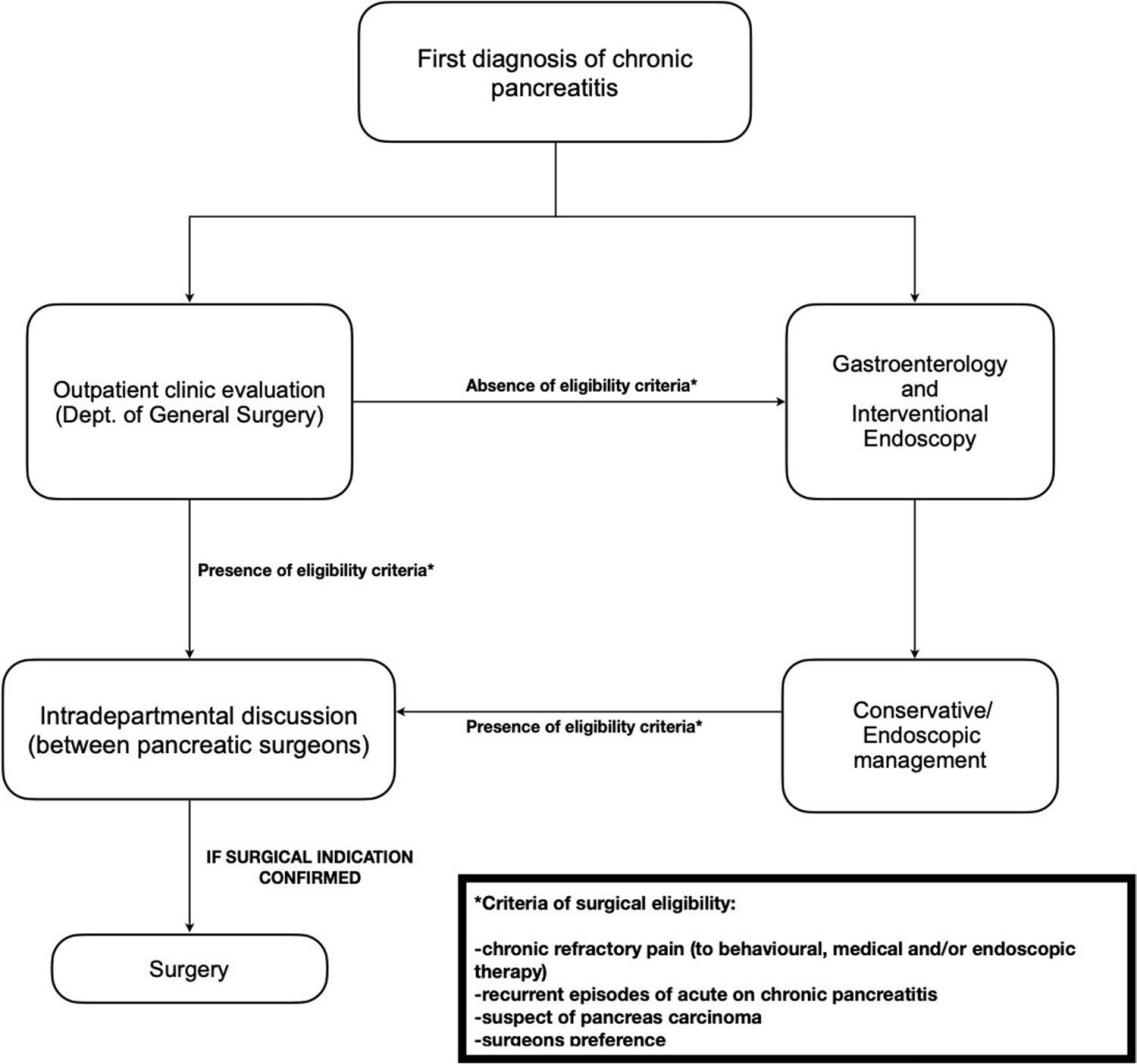
Selection criteria for surgery for CP at the Vienna General Hospital

**Fig. 2:**
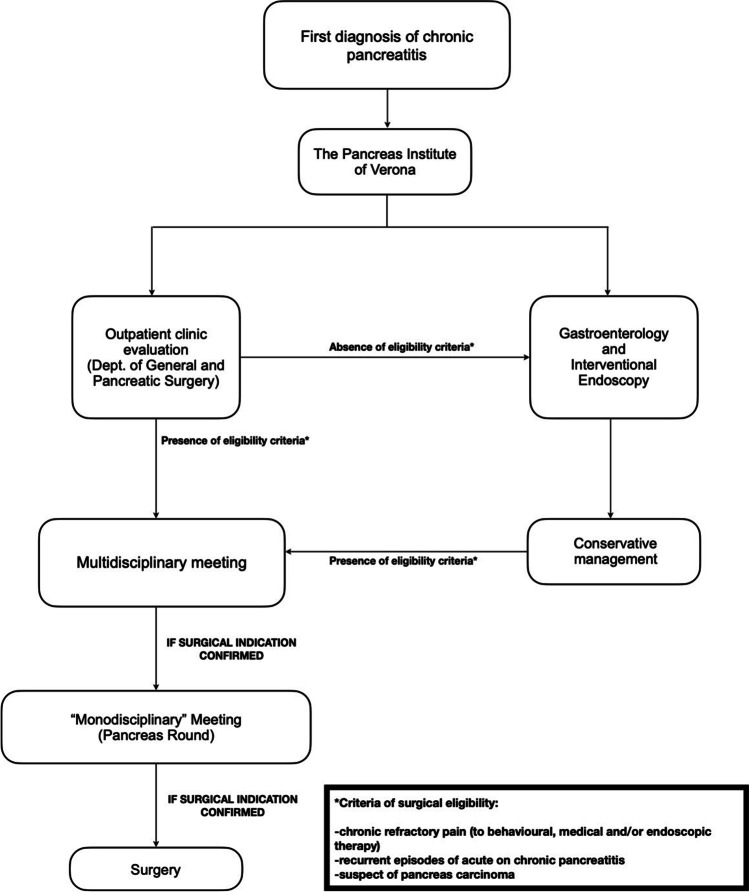
Selection criteria for surgery for CP at the Verona University Hospital

**Fig. 3:**
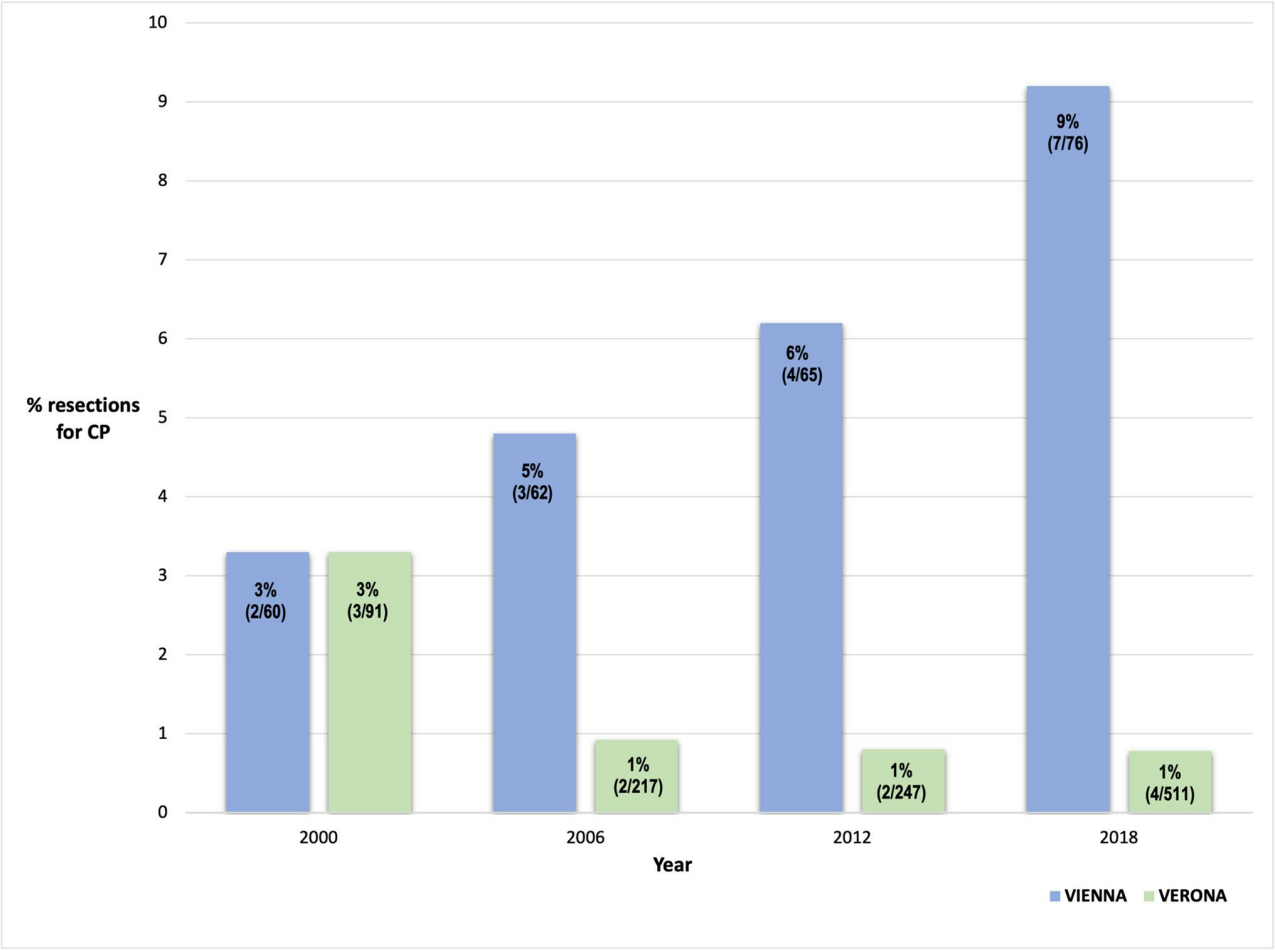
Evolution over time of surgery for chronic pancreatitis at the two centers involved

Between 2000 and 2018, a total of 168 patients underwent surgery for CP, 77 at the Department of General Surgery of Vienna, and 91 at the Department of General and Pancreatic Surgery of Verona. The flowcharts reported in Figs. 1 and 2 outline the clinical management of CP and the eligibility criteria for surgery at the two institutions considered. Table 1 details the main general characteristics of the Italian and Austrian populations. In both series, most of the included patients were men (75% vs 85%, *p* = 0.131) aged around 50 (48.3 vs 50.8, *p* = 0.425, *d* = 0.218 [CI − 0.52–0.08]). No significant differences were detected between the two cohorts in terms of comorbidities, BMI, risk factors for CP, and presenting symptoms (Table 1). The analyzed data showed a considerable progressive decrease in the annual rate of pancreatic surgical procedures for CP during the 2000–2018 period at the Department of General and Pancreatic Surgery of Verona (from 3% to less than 1%); by contrast, the percentage of surgeries for CP performed at the Vienna General Hospital increased from 3 to 9% during the same time frame (Fig. 3). More than 70% of patients at both centers (77% in Vienna, 71% in Verona, *p* = 0.420) underwent at least one attempt at endoscopic treatment before surgery (median 2 [IQR 2.0] at the Department of Surgery of Vienna, 2 [IQR 3.1] at the Department of General and Pancreatic Surgery of Verona, *p* = 0.985). The median time span between first diagnosis and surgical intervention amounted to four years (IQR 5.5) in the Austrian series and two years (IQR 4.0) in the Italian series (*p* = 0.048).

**Table 1:** General features

	Vienna *n* = 77	Verona *n = 91*	*p* value
**Sex [no. (%)]**			
M	58 (75)	77 (85)	0.131*
F	19 (25)	14 (15)	
**Age at diagnosis, mean (± SD, years)**	48.3 (± 9.8)	50.8 (± 12.7)	0.425**
**ACCI [median (IQR)]**	2 (2)	1 (2)	0.158***
**BMI [median (IQR)]**	21.6 (4.2)	21.2 (4.7)	0.726***
**Smoker [no. (%)]**	58 (75)	66 (73)	0.737*
**Ex-smoker [no. (%)]**	8 (10)	12 (13)	0.198*
**Alcohol consumption [no. (%)]**	45 (58)	48 (53)	0.107*
**Ex-alcohol consumption [no. (%)]**	18 (23)	18 (20)	0.683*
**Familiarity for chronic pancreatitis [no. (%)]**	3 (4)	6 (7)	0.439*
**Genetic mutations [no. (%)]** **(CFTR, SPINK 1 or both)**	1 (1)	4 (4)	0.239*
**Anatomic abnormalities of the pancreas [no. (%)]**	6 (8)	4 (4)	0.354*
**Diabetes mellitus or glucose intolerance, [no. (%)]**	16 (21)	11 (12)	0.220*
**Pancreatic exocrine impairment [no. (%)]**	36 (47)	19 (21)	0.075*
**Symptoms [no. (%)]**			
Recurrent or chronic pain	67 (87)	75 (82)	0.129*
Acute on chronic pancreatitis	31 (40%)	37 (41)	0.958*
Tiredness and lack of energy	2 (3)	8 (9)	0.091*
Dyspepsia	7 (9)	17 (19)	0.077*
Impaired bowel function	7 (9)	6 (7)	0.546*
Jaundice	6 (8)	11 (12)	0.358*
Weight loss	19 (25)	38 (42)	0.020*

*SD* standard deviation, *IQR* interquartile range, *ACCI* age-adjusted Charlson comorbidity index, *BMI* body mass index, *CFTR* Cystic fibrosis transmembrane conductance regulator, *SPINK 1* serine protease inhibitor Kazal-type 1

Statistical analysis: * = χ2 test; ** = *t*-Student test; *** = Wilcoxon test

Table 2 reports the main differences between the two cohorts in terms of clinical suspicion, surgical techniques, and postoperative course. According to the institutional medical reports, the generical definition “chronic pancreatitis” was the most frequently reported preoperative clinical diagnosis at both centers (97% in Vienna vs 79% in Verona); nevertheless, the rate of clinically and/or radiologically suspected groove pancreatitis was sensibly higher in the Verona cohort (18% vs 3%, *p* = 0.005).

**Table 2:** Surgery and post-operative course

	Vienna *n* = 77	Verona *n* = 91	*p* value
**Preoperative clinical suspicion [no. (%)]**			
Chronic pancreatitis	75 (97)	72 (79)	0.005*
Groove pancreatitis	2 (3)	16 (18)	
PDAC	0	2 (2)	
Pancreatic cyst(s)	0	1 (1)	
**Type of surgery [no. (%)]**			
Resective (PPPD, Whipple-PD, distal or total pancreatectomy)	55 (71)	83 (91)	0.005*
Decompressive/drainage (Frey or Partington-Rochelle)	22 (29)	8 (9)	
**Open vs VLS [no. (%)]**			
Open	76 (99)	91 (100)	0.276*
VLS	1 (1)	0	
**Postoperative complications (overall) [no. (%)]**	26 (34)	30 (33)	0.913*
POPF	12 (16)	10 (11)	0.379*
DGE	0	3 (3)	0.108*
PPH	6 (8)	7 (8)	0.750*
Biliary fistula	1 (1)	5 (5)	0.144*
Abdominal collections	7 (9)	13 (14)	0.300*
Other	15 (19)	14 (15)	0.484*
Reintervention	7 (9)	3 (3)	0.114*
**Perioperative mortality [no. (%)]**	1 (1)	0	0.276*
**Hospital stay (days, median, IQR)**	10 (6.5)	8 (6.2)	0.004***
**Histology [no. (%)]**			
Chronic pancreatitis	75 (97)	77 (85)	0.005*
Groove pancreatitis	2 (3)	14 (15)	

*IQR* interquartile range, *PDAC* pancreatic ductal adenocarcinoma, *PPPD* pylorus-preserving pancreatoduodenectomy, *VLS* videolaparoscopy, *POPF* postoperative pancreatic fistula, *DGE* delayed gastric emptying, *PPH* post-pancreatectomy hemorrhage

Statistical analysis: * *χ*^2^ test; *** Wilcoxon test

Although the resective procedures represented the predominant surgical strategy at both Institutions, a significant difference in the number of decompressive/drainage operations performed was detected (29% in Vienna vs 9% in Verona, *p* = 0.005).

The overall rate of postoperative morbidity was comparable between the two groups (34% vs 33%, *p* = 0.913), as well as the postoperative mortality (1% vs 0, *p* = 0.276). Likewise, the analysis of the single postoperative complications did not point out any significant inter-institutional differences. The median length of hospital stay was 10 (IQR 6.5) days at the Department of General and Pancreatic Surgery of Verona and eight (IQR 6.2) days at the Department of Surgery of Vienna (*p* = 0.004). CP was the predominant definitive diagnosis in both groups (97% and 85%). Despite this, the rate of histologically proved groove pancreatitis was significantly higher in the Italian series (15% vs 3%, *p* = 0.005); in this regard, the term “groove pancreatitis” was reported in less than the half of the pathological reports (no. = 1 in Vienna, no. = 6 in Verona), while in the other cases a range of different synonyms (e.g., paraduodenal pancreatitis, duodenal dystrophy, duodenal wall cysts, pancreatic amartoma of the duodenum) were detected.

## Discussion

The results of this retrospective analysis emphasized two different institutional policies towards the surgical treatment of chronic pancreatitis. In fact, although the candidates for surgery displayed similar demographic and clinical characteristics at both centers, considerable inter-institutional differences were found in terms of frequency, timing, and type of the operative strategies. In the context of contemporary surgical reality, based on the standardization of treatments and on the selection of best evidence-based therapeutical approach for each specific pathology [10], such results should be reviewed and analyzed in great detail. This is, ultimately, the main purpose of the present study.

In line with the data reported in literature [11], both patient cohorts mostly consisted of men aged around 50 and generally included patients presenting with few comorbidities (median age-adjusted Charlson Comorbidity Index [ACCI] 1–2, see Table 1). According to the M-ANNHEIM Classification, CP results from the synergic interaction of multiple risk factors, which can be grouped into two major categories, namely the biological predisposition and the environmental causes [12]. Within this last group, the most prevalent etiological factors are represented by smoking and alcohol consumption [1]. In fact, more than 85% of patients in our two series were heavy smokers or ex-smokers with an average consumption of 20 cigarettes/day (20 [IQR 15.0] in the Austrian group, 20 [IQR 11.2] in the Italian group, *p* = 0.624). Unexpectedly, the rate of patients with a previous or actual history of habitual alcohol consumption ≥ three alcoholic units/day was relatively low (about 20% and 50%, respectively). The most probable explanation behind these findings is that in these past few years heavy alcohol intake appears to have lost its almost exclusive etiological role in the development of CP, due to the increased identification of nosological entities - previously often misdiagnosed - mimicking CP (e.g., hereditary pancreatitis, autoimmune pancreatitis, and groove pancreatitis) [13], and to the significant changes in many demographical and cultural factors - e.g., alcohol quality and type of beverages consumed [14] - that have occurred over the time.

Chronic or recurrent abdominal pain represented the leading clinical manifestation in both cohorts (87% vs 82%, *p* = 0.129), followed by recurrent episodes of acute on chronic pancreatitis (40% vs 41%, *p* = 0.958). In total, only 2% of patients (three out of 168) did not exhibit relevant clinical symptoms and were allocated to surgery due to the suspicion of malignancy (no. = 2) or of a mucinous pancreatic cystic neoplasia (no. = 1). These percentages essentially reflect the evidence reported in literature [15], although they could be influenced by the selection criteria of our cohort (“surgical series”), as chronic pain commonly represents the main indication for surgery [16].

The observed overall rate of postoperative morbidity (approximately 33% at both centers) and postoperative mortality at 90 days (< 2%) were aligned with - or possibly lower than - what is reported in current literature with regard to pancreatic surgery in highly specialized centers [17]. However, considering the benign nature of CP and the low rate reported for endotherapy-associated morbidity [18], these percentages must not be underestimated and the potential complications of surgery should always be taken into account during the therapeutical decision-making process.

Despite all the similarities in terms of baseline and clinical patient characteristics outlined above, the comparison between the surgical managements adopted at the two centers revealed many remarkable discrepancies. In order to adequately analyze such inconsistencies, a grasp of the actual role of surgery in clinical practice is crucial. The therapeutical indications for chronic pancreatitis have changed significantly over the past decades [7]. During the second half of twentieth century, a plethora of pioneering and sometimes revolutionary surgical procedures for the treatment of CP were described [19]. As reported by Pederzoli et al*. *[20], at the beginning of the 90s up to 60% of patients affected by CP-related chronic pain underwent surgery throughout their lifetime, proving that this approach was key in the treatment of this pathology at the time. From the 2000s onwards, a radical change of direction in the management of CP was observed and the various conservative approaches (pharmacological therapy and interventional endoscopy) assumed an increasingly first-line role in the management of CP; as a result, surgical therapy became the last treatment option for unresponsive symptomatic patients [21, 21]. Nevertheless, the feasibility, applicability, and timing of the surgery are still the subject of debate in the surgical community today [2].

Despite the recent efforts to provide the clinical practitioners with a set of evidence-based recommendations aiming at standardizing and guiding every step of the diagnostic and therapeutic process in CP patients [23, 23], an acceptable level of adherence to these guidelines seems far from being achieved [25]. Accordingly, our retrospective analysis showed two opposite trends in the adoption of surgery for CP over the last 20 years (Fig. 3): whereas the number of surgical interventions for CP progressively decreased in the Italian series (from 3% to less than 1%), it increased considerably in the Austrian series (from 3 to 9%). Furthermore, the time-lapse between first diagnosis and surgery vastly differed between the two cohorts (approx. four years in Vienna, approx. two years in Verona). Based on these data, two different types of surgical strategy can be delineated: (i) an increasingly conservative policy tending to anticipate surgery whenever indicated (the “Verona approach”); (ii) a more frequent adoption of surgery, but with longer preoperative observational periods (the “Vienna approach”). The reasons behind such discrepancies - which appear even more surprising considering that the two centers shared the same selection criteria for surgery (Figs. 1 and 2) - are likely to be diverse and difficult to interpret. Our prevailing hypothesis is that the choice of operative strategy, as well as of its performance, are deeply influenced by the historical background and the consolidated internal policy of each surgical center. Even if the widespread “devotion” to the diktats of “surgical schools” and the personal beliefs of physicians often hinder the implementation of changes in surgical practice, the availability of a valid and shared set of globally accepted recommendations - that, also in this regard, are still lacking - could probably mitigate this phenomenon and lead to a drop in inter-institutional treatment disparities [26, 26]. Moreover, it has been proved that many regional variations in clinical decision-making are influenced by an assortment of factors varying greatly across countries, such as the degree of involvement of patients in treatment decisions, the regional intensity of medical care, the amount of financial incentives, and the expertise of surgeons [28]. In this context, it is worth to be mentioned that the two Institutions involved in this study - although they are both definable as “high-volume centers” [17] - present substantial differences in terms of patient volume and “degree of specialization”. Indeed, as depicted in Figs. 1 and 2, the preoperative diagnostic process in Vienna appeared to be more dependent on the subjective clinical decisions than in Verona, where the existence of a totally dedicated interdepartmental working group - namely, the Pancreas Institute - probably guided the therapeutical decisions following a more comprehensive and multidisciplinary perspective.

Further significant inter-institutional differences were also observed with regard to the preoperative clinical suspicion and the definitive histological diagnosis (Table 2). Specifically, the number of patients with a suspected groove pancreatitis (GP) was considerably higher in the Verona series (18% vs 3%, *p* = 0.005), as well as the rate of confirmed GP (15% vs 3%, *p* = 0.005) at the definitive histopathological examination of the surgical specimen. There are a number of explanations that could be responsible for this deviation. In fact, the preoperative diagnosis of GP still represents a challenge for clinicians, as this pathology belongs to the heterogeneous group of “inflammatory/tumour-like lesions” of the pancreas: as suggested by their name, these disorders can mimic a cancer or another malignant neoplasm of the pancreatic gland, leading to a certain rate of misdiagnosis and, consequently, to under- or overtreatment [29]. The preoperative radiological diagnosis of GP could prove extremely arduous - even in specialized centers - for untrained radiologists, and its subtle, often unspecific histological features make preoperative sampling not useful or confusing at times [30]. In addition, a unanimous agreement about the therapeutical management of GP has not yet been reached. Whereas many authors uphold the high efficacy and suitability of conservative treatments [30], other studies clearly promote the application of a resective policy due to its effectiveness in achieving symptom relief [31, 31]. The Verona experience - recently reported by Balduzzi et al*. - *showed similar results in the comparison between operative and medical therapy in terms of quality of life and pain control, therefore fostering the adoption of surgery - which is potentially burdened by relevant complications - only in appropriately selected cases and following careful multidisciplinary risk-benefit assessment [33, 33]. Lastly, the revision of the postoperative pathology reports performed upon data collection uncovered - especially in the past - semantic confusion in the codification of this disease. Indeed, aside from the abundance of definitions used for GP (e.g., paraduodenal pancreatitis, cystic dystrophy, heterotopic pancreas, duodenal wall cysts etc.) [35], many generic, unclear, and sometimes misleading descriptions were detected at both centers. Accordingly, the real incidence of this pathology is still controversial. In order to accurately determine it, a systematic review of the surgical specimens - that in this context, due to the long study period, could not be performed - , as well as the conduction of multicentric studies on a larger scale are needed. Once again, all these considerations point out that the lack of structured common benchmarks and recommendations could potentially lead to significant diagnostic and treatment disparities.

This study has several limitations, including its retrospective design. Due to the long study period and the incompleteness of many relevant clinical and surgical data, the results were compromised. In order to mitigate this bias, the data were accurately reviewed and extended throughout the anamnestic and clinical information reported in the postoperative outpatient clinic evaluations. For the same reason, the authors were unable to perform an in-depth comparative analysis of the surgical indications given at the two centers. Moreover, in the absence of adequate information about the long-term postoperative course, the impact of surgery on patients’ health status and quality of life could not be appraised; further studies are required to perform a comprehensive analysis of this topic.

## Conclusion

The comparison of the surgical attitude towards chronic pancreatitis between two high-volume centers proved that a uniform approach to this pathology still is lacking. Although surgery can be carried out with satisfactory results in specialized centers, its adoption should always be given careful consideration as it should be tailored to specific clinical scenarios and single individuals. In this regard, the identification of strict criteria and labels of surgical eligibility is advisable, and further efforts should be made by the surgical and gastroenterological community to designate a set of management protocols and strategies acknowledged on a global scale.

## Data Availability

Not applicable.
